# The Convergence of Biotechnological Strategies in Medicinal Plants: From Individual Approaches Toward an Integrated Systems Framework

**DOI:** 10.3390/ijms27167407

**Published:** 2026-08-19

**Authors:** Katarzyna Hnatuszko-Konka, Aneta Gerszberg, Marta Libik-Konieczny, Monika Tuleja, Grzegorz Góralski, Monika Bojko, Magdalena Kędra, Beata Myśliwa-Kurdziel, Paweł Kowalczyk, Aneta Wiktorek-Smagur, Mariusz Krupiński

**Affiliations:** 1 Department of Molecular Biotechnology and Genetics, Faculty of Biology and Environmental Protection, University of Lodz, Banacha 12/16, 90-237 Lodz, Poland; 2 The Franciszek Górski Institute of Plant Physiology, Polish Academy of Sciences, Niezapominajek 21, 30-239 Kraków, Poland; 3 Department of Plant Cytology and Embryology, Institute of Botany, Faculty of Biology, Jagiellonian University in Kraków, Gronostajowa 9, 30-387 Kraków, Poland; 4 Department of Plant Physiology and Biochemistry, Faculty of Biochemistry, Biophysics and Biotechnology, Jagiellonian University in Kraków, Gronostajowa 7, 30-387 Kraków, Poland; 5 Department of Animal Nutrition, The Kielanowski Institute of Animal Physiology and Nutrition, Polish Academy of Sciences, Instytucka 3, 05-110 Jabłonna, Poland; 6 The Academy of Business and Health Sciences, Piotrkowska 278, 90-361 Lodz, Poland; 7 Department of Industrial Microbiology and Biotechnology, Faculty of Biology and Environmental Protection, University of Lodz, Banacha 12/16, 90-237 Lodz, Poland

**Keywords:** medicinal plants, bioactive compounds, plant tissue culture-based biotechnology, metabolic engineering, pharmaceuticals, multi-omics, bioinformatics, nanotechnology, unconventional elicitors

## Abstract

Medicinal plants remain one of the most important sources of therapeutically relevant compounds for pharmaceutical, nutraceutical, and biotechnological applications. However, the naturally low abundance of many specialized metabolites, considerable phytochemical variability, and increasing environmental pressures continue to limit the sustainable exploitation of plant-derived bioactive compounds. This review summarizes current strategies aimed at identifying, understanding, and enhancing the production of medicinally valuable metabolites while highlighting both biological limitations and emerging technological opportunities. Particular attention is given to the interplay between primary and secondary metabolism and its role in determining biosynthetic efficiency in both natural and engineered systems. Beyond discussing established methodologies, this review adopts a broader perspective by incorporating less frequently addressed aspects, including the influence of climate change on metabolite production, the application of bioinformatics-supported approaches to improve bioprospecting efficiency, and the growing role of nanoparticles as elicitors in plant biotechnology. These topics are considered alongside advances in tissue culture technologies, metabolic engineering, molecular approaches, and systems-level analyses aimed at improving metabolite yield and production stability. Current evidence suggests that no single technological framework is sufficient to address the complexity of medicinal plant metabolism. Hence, rather than presenting individual technologies as isolated solutions to specific biosynthetic bottlenecks, this review emphasizes that medicinal plant metabolism should be considered a highly interconnected system in which environmental, molecular, and physiological factors collectively determine production outcomes.

## 1. Introduction

The perception and exploration of plants has have changed significantly as a result of exceptional advances in biotechnology. Plants have been used for thousands of years in agriculture and herbalism, and in modern times their importance has grown since plant material has been applied for various industrial purposes and medicines. Coined in 1967 during studies on hallucinogenic plants, the term “medicinal plants” refers to species whose various parts (including flowers, leaves, roots, seeds, etc.) are employed either directly or as ingredients in formulations for the treatment of diseases and health conditions [1]. Medicinal plants are among the oldest forms of medicine and have a long history of use in traditional healthcare systems worldwide. Yet, despite major advances in modern medicine, nearly 80% of the global population continues to rely on traditional herbalism, reflecting both its deep roots in cultural traditions and the unequal availability of modern pharmaceutical therapies [2]. This observation is particularly striking in light of current demographic trends. According to the most recent real-time, dynamic estimates elaborated by Worldometer, the current world population reached approximately 8.27 billion in 2026 [3]. In this context, the continuous search for new bioactive compounds is driven by the increasing prevalence of chronic and infectious diseases, the emergence of drug-resistant pathogens, and the ongoing demand for safer, more effective therapeutics. Another current challenge for humanity is climate change, which influences plant physiology and consequently the synthesis of required substances. Therefore, it emerges as a determinant that can be either beneficial or detrimental, affecting their production [4]. Additionally, the climate-driven modification of habitat suitability and geographical distribution of medicinal plants may have implications for commercial supply chains that depend on region-specific plant resources. This is because variability in bioactive compound composition affects not only the efficacy and safety of herbal products but also the reproducibility required for pharmaceutical and nutraceutical applications [5,6]. The challenges outlined above, together with the continuing importance of medicinal plants in healthcare, are also reflected in recent international initiatives. In 2025, the World Health Organization (WHO) launched the first global Traditional, Complementary and Integrative Medicine (TCIM) dashboards, covering all 194 WHO Member States as part of the WHO Global Traditional Medicine Strategy 2025–2034. This unprecedented initiative highlights the growing recognition of medicinal plant resources and traditional medicine as strategic components of contemporary healthcare systems while providing a harmonized framework for monitoring national policies and implementation [7].

Against this background, the continued global interest in medicinal plants has further reinforced the importance of exploring their largely untapped chemical diversity. Medicinal plants constitute a vast and still largely unexplored reservoir of chemically diverse natural products, making them a central focus of modern bioprospecting and natural product research. Natural products continue to play a major role in modern drug discovery, with compounds of purely synthetic origin accounting for only about 36% of currently used pharmaceuticals [8]. In this context, bioprospecting is the systematic exploration of biological diversity to identify chemical (compounds), genetic (DNA/genes of desirable traits for applications in biotechnology), and bionic (biomimetic designs of processes or structures) resources with potential scientific, industrial, or commercial value. In the case of medicinal plants, bioprospecting mainly focuses on discovering bioactive molecules that can serve as leads for drug development or other therapeutic applications [9,10]. Combining insights from traditional medicine with the study of genetic convergence and the latest advances in bioinformatics can lead to the discovery of entirely new products or reveal novel uses for already known compounds [11]. A clear historical example of such targeted bioprospecting efforts is provided by large-scale anticancer screening programs initiated in the mid-20th century. Coordinated initiatives started by the Cancer Chemotherapy National Service Center (CCNSC, USA) were launched to evaluate natural resources as a source of bioactive compounds. Approximately 115,000 extracts were collected and systematically analysed, yielding the discovery of the natural diterpene anticancer agent, paclitaxel *(Taxus brevifolia)*. Today, this compound is widely used as an antineoplastic drug in the treatment of various human cancers [12,13]. Similarly, numerous widely used therapeutics that originated from natural sources, including aspirin (*Salix* sp.) or the antimalarial quinine (*Cinchona* sp.), were successfully introduced to clinical use through this approach [14].

The prominent role of natural sources in drug development is unsurprising, as plant-derived secondary metabolites exhibit diverse therapeutic properties, including anti-inflammatory, antioxidant, anticancer, antibacterial, and hepato- and neuroprotective effects, many of which underpin current pharmacological treatments. However, medicinal plants produce only small, often unstable amounts of these compounds (the fundamental bottleneck in pharmacognosy), with concentrations influenced by growth conditions, genotype, and developmental stage [15,16,17]. In this context, solutions based on unsustainable collection of wild plants can significantly disrupt ecosystems and negatively impact environmental balance. On the other hand, the chemical synthesis of secondary bioactive metabolites at an industrial level remains economically and technically challenging due to their complex chemical structures [18]. Hence, these constraints set limits on meeting continuously growing market demands, underscoring the need to enhance natural biosynthetic rates of bioactive compounds. To eliminate this bottleneck, two main types of approaches can be considered: increasing the synthesis level through plant tissue-culture-based biotechnology or exploiting the methods of widely understood biotechnological engineering (including metabolic engineering, tissue culture engineering, and gene editing technologies). Most importantly, biotechnology enables the production of these compounds in a controlled, year-round manner, ensuring consistent yields while providing the means to make process scale-up possible and, in many cases, more feasible than conventional approaches. In addition, it facilitates downstream handling, making the extraction and purification of target metabolites more straightforward compared with conventional plant-based methods. Moreover, the observed advances in genetic engineering, tissue culture, ‘omics’ technologies, bioinformatics, and nanotechnology have opened new pathways for a more complete and precise exploitation of the therapeutic potential of medicinal plants. In particular, high-throughput omics datasets derived from genomics, transcriptomics, proteomics, and metabolomics provide a robust foundation for mechanistic insights into drug discovery and therapeutic repurposing [19]. It should be noted that, although the biotechnological strategies discussed in this review may influence the broader phytochemical profile of medicinal plants, the present review focuses primarily on therapeutically relevant plant-derived secondary metabolites, which constitute the principal group of bioactive compounds considered throughout this article.

To illustrate the challenges and opportunities outlined above, Table 1 summarizes representative medicinal plant species together with their principal bioactive secondary metabolites, therapeutic applications, and the biotechnological strategies developed to overcome current limitations in metabolite production.

Improving specialized metabolism requires intervention in highly interconnected regulatory networks that link primary and secondary metabolism across multiple biological levels. Against this background, the following sections discuss the principal biotechnological strategies currently applied in medicinal plant research. These strategies range from tissue-culture-based approaches and elicitation, through genetic and metabolic engineering, to omics technologies, bioinformatics, and artificial intelligence. This organization reflects both the historical development of the field and the progressively increasing depth at which these technologies enable investigation and manipulation of plant metabolism. However, it should not be interpreted as a succession of competing methodologies or as a replacement of earlier approaches by newer ones. Rather, each technological advance has provided access to additional regulatory layers, progressively revealing the complexity of medicinal plant metabolism and expanding the possibilities for its rational manipulation.

**Figure 1:**
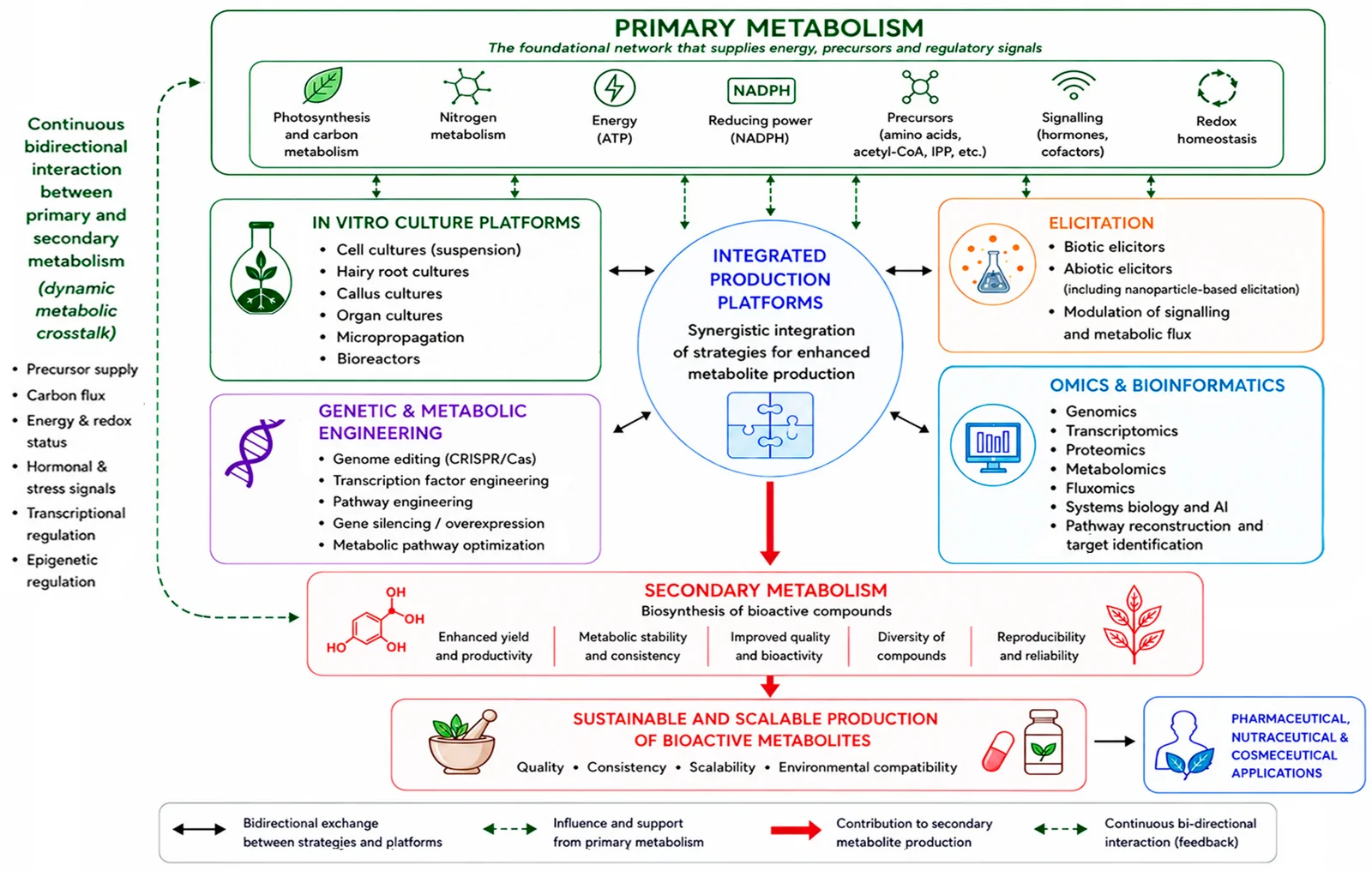
Conceptual framework illustrating the systems-oriented perspective in this review. Primary metabolism supplies energy, precursors, reducing power, and regulatory signals for secondary metabolite biosynthesis. The principal biotechnological strategies (in vitro culture, elicitation and nanoparticles, genetic and metabolic engineering, and omics and bioinformatics) are presented as complementary approaches at different regulatory levels. Together, they contribute to the efficient and sustainable production of bioactive compounds. The scheme highlights the bidirectional interaction between primary and secondary metabolism, emphasizing that understanding metabolic complexity supports the integration of complementary biotechnological strategies.

Accordingly, this review proposes a conceptual perspective in which these individual strategies are considered within the broader context of an interconnected metabolic system. Whether continued technological development will ultimately lead to broadly integrated, systems-level solutions remains an open question. Nevertheless, the growing recognition of this biological complexity increasingly points to the integration of different levels of intervention as a logical consequence of current progress and provides the conceptual framework through which the topics discussed in this review should be interpreted (Figure 1).

## 2. Influence of Primary Metabolic Pathways on Bioactive Metabolite Production in In Situ and In Vitro Culture

Plant metabolism comprises an intricate network of biochemical reactions essential for plant physiology, growth, and development. It is broadly divided into primary and secondary metabolism. Primary metabolism produces fundamental compounds such as carbohydrates, amino acids, nucleotides, and lipids. Secondary metabolism yields structurally diverse bioactive compounds, including alkaloids, phenolics such as flavonoids and tannins, terpenoids, glycosides (including saponins and cyanogenic glycosides), polyketides, and sulfur-containing compounds. Together, these represent the major classes of plant secondary metabolites [52]. While primary metabolites are indispensable for plant survival, secondary metabolites (hereafter referred to interchangeably as specialized metabolites) primarily fulfil ecological functions, although they also represent the predominant source of therapeutically active plant compounds. These include defence against pathogens, herbivores, and abiotic stress (e.g., UV radiation, drought, and temperature extremes), attraction of pollinators and seed dispersers, allelopathic interactions with competing plants, as well as functioning as signalling molecules in plant–environment interactions [53].

In vitro culture systems (whole plants, plant organs, cell suspensions) provide a versatile and sustainable platform for producing bioactive metabolites. Their efficiency depends on both optimised culture conditions and a comprehensive understanding of primary and secondary metabolic pathways. Dedifferentiated plant cells in vitro exhibit marked metabolic plasticity, enabling the redirection of metabolic fluxes through controlled manipulation of culture parameters [54]. Due to controllable and reproducible conditions, these systems bypass constraints related to geography, seasonality, plant developmental stages, and climate-driven alterations in plant physiology and metabolism. Furthermore, plant in vitro cultures provide the biological foundation for the tissue-culture-based biotechnological approaches discussed in the following section.

Although secondary metabolism produces the structurally diverse bioactive compounds of interest, it depends on primary metabolism for energy and precursors, particularly photosynthesis, respiration, and redox/antioxidant systems. Understanding these interdependencies is crucial for designing strategies to enhance metabolite production [55]. Photosynthesis supplies fixed carbon and reducing power in the form of reduced nicotinamide adenine dinucleotide phosphate (NADPH) and adenosine triphosphate (ATP), which drive biosynthetic reactions. While most in vitro systems rely on sucrose as a carbon source, photoautotrophic or photomixotrophic cultures can activate plastidial metabolism and enhance flux through pathways such as the shikimate pathway (for phenolics and many alkaloids) or terpenoid biosynthetic pathways. Light intensity and spectral quality strongly influence photosynthetic activity and, consequently, secondary metabolite accumulation [56,57]. Cultures with enhanced photosynthetic capacity often accumulate higher levels of phenolic antioxidants, flavonoids, and carotenoids.

Mitochondrial respiration provides ATP and metabolic intermediates (acetyl-CoA, α-ketoglutarate, malate) that serve as precursors for many secondary metabolic pathways. High respiratory activity may support the production of nitrogen-containing secondary metabolites (alkaloids, glucosinolates), which require carbon-nitrogen integration through the tricarboxylic acid (TCA) cycle and amino acid metabolism. In vitro stress from elicitors (see below) or osmotic shifts, frequently elevates respiration, which can redirect carbon flux from growth to secondary metabolism. These primary metabolic pathways are tightly interconnected [52,58]. Carbohydrate metabolism via glycolysis and the oxidative pentose phosphate pathway supply essential precursors for secondary metabolism. Specifically, these processes generate molecules, which supply shikimate pathway for the synthesis of aromatic secondary metabolites. Photosynthetic carbon fixation in plastids increases sugar availability, which enhances mitochondrial respiration and the supply of carbon skeletons for secondary metabolism. Furthermore, ATP and NADPH from photosynthesis and reduced nicotinamide adenine dinucleotide (NADH) from respiration drive energetically demanding steps of secondary metabolite biosynthesis, such as hydroxylation and glycosylation [17,59,60].

Because of these tight metabolic linkages, particularly those involving photosynthesis and mitochondrial respiration, manipulating primary metabolic fluxes can strongly influence secondary metabolite production. These relationships further indicate that isolated interventions targeting single pathways may provide only limited improvements. Greater potential lies in the coordinated manipulation of environmental, metabolic, and regulatory factors to achieve stable enhancement of specialized metabolite production. Several culture-related factors are known to exploit these metabolic relationships. These include nutrient composition, plant growth regulators, elicitors, and physical culture conditions such as light quality and intensity, temperature, pH, and osmotic status. Together, these factors influence enzyme activities and the expression of genes involved in secondary metabolism [57].

Some of these phenomena known from in situ observations demonstrate that climate change exerts a significant influence on the production of secondary metabolites in medicinal plants. Importantly, these responses are highly species- and compound-specific, resulting in considerable variability in phytochemical profiles [4]. Under natural growth conditions, rising temperatures, altered precipitation patterns and soil conditions, increased atmospheric CO_2_ levels, and the growing frequency of extreme weather events collectively influence phytochemical composition, modulating metabolic pathways involved in the biosynthesis of therapeutically relevant metabolites [61]. The levels of certain bioactive compounds may be reduced by high temperatures alone as a consequence of metabolic adjustments that prioritize survival over defence mechanisms [62,63,64]. Elevated temperatures and increased CO_2_ availability can promote vegetative growth or biomass accumulation in some medicinal plants, without a guaranteed improvement in phytochemical composition. As key drivers of climate change, both factors directly or indirectly, via drought and other extreme weather events, exert contrasting effects on plant metabolism. They may enhance the accumulation of some metabolites while constraining others because of complex carbon partitioning and temperature-dependent metabolic regulation. For instance, in *Melia azedarach*, water deficit conditions resulted in an increase in the concentration of both phenols and flavonoids, whereas the biosynthesis of salvianolic and coumaric acids in *Lavandula latifolia* plants under drought conditions was reduced [65,66]. Similarly, experimental evidence from *Brassica oleracea* L. demonstrates that exposure to low or high temperatures can markedly alter phytochemical profiles, thereby modifying the biological activity of plant extracts. Low-temperature conditions promote the accumulation of defense-related metabolites such as phenolics and glucosinolates, whereas high temperatures may suppress specific secondary metabolites while enhancing osmolyte production [67]. Consequently, based on the premise that moderate stress conditions can stimulate the accumulation of defense-related metabolites as part of adaptive stress responses, controlled abiotic stressors are used as triggers in elicitation systems. Notably, observations of climate-induced metabolic responses in plants therefore serve primarily as a conceptual framework for hypothesis generation rather than a direct blueprint for laboratory conditions. Nevertheless, these adaptive metabolic shifts provide a valuable starting point for designing and refining laboratory-based cultivation and elicitation strategies aimed at enhancing the production of targeted bioactive compounds.

The above strategies have successfully enhanced the production of high-value phytochemicals such as artemisinin (from *Artemisia annua*) and other diverse phenolic antioxidants from various medicinal plant species [52,68]. In addition, the production of secondary metabolites, especially phenolic antioxidants, flavonoids, and terpenoids, is frequently triggered by oxidative stress. In vitro conditions often induce reactive oxygen species (ROS), which act as signalling molecules to activate secondary metabolism. The antioxidant defense system (superoxide dismutase, catalase, ascorbate-glutathione cycle) regulates ROS levels and influences metabolic gene expression [69]. Moderate ROS accumulation can activate transcription factors (TF), including MYB-bHLH-WD40 (MBW) complexes, which regulate structural genes encoding phenylalanine ammonia-lyase (PAL), cinnamate 4-hydroxylase (C4H), and 4-coumarate-CoA ligase (4CL) in the phenylpropanoid pathway. Conversely, excessive ROS can suppress biosynthesis and damage cells, highlighting the need for strict redox homeostasis [70,71].

Collectively, these biological relationships constitute the mechanistic basis for current biotechnological strategies aimed at enhancing secondary metabolite production. The following sections describe how individual approaches exploit different levels of this metabolic regulation.

## 3. Tissue-Culture-Based Biotechnology for Bioactive Compound Production

Among other factors, the understanding of the previously highlighted stress-induced metabolism underpins the development of effective tissue culture technology. The tissue-culture-based biotechnology approach relies on a range of genetic modification-independent techniques aimed at boosting the production of bioactive compounds. Though they do not involve direct genetic modification, they enable efficient large-scale propagation of medicinal plants under limited spatial and temporal constraints and support long-term conservation and storage. At the same time, they provide a fundamental starting point for subsequent genetic transformation studies and the production of valuable metabolites. Unequivocally, knowledge of the interdependencies between primary and secondary metabolic pathways enables targeted manipulation of metabolic fluxes toward secondary metabolite biosynthesis. Importantly, tissue culture systems should not be viewed solely as independent production platforms but rather as experimental frameworks in which cultivation parameters, elicitation regimes, precursor supply, and molecular interventions can be integrated and iteratively optimised. Moreover, plant tissue culture provides an efficient in vitro strategy for obtaining genetically uniform, virus-free plant material. By culturing selected explants such as meristems or shoot tips, often combined with thermal or chemical treatments, this approach improves plant vigour, yield potential, and overall biological quality, resulting in measurable agronomic and economic benefits [72].

As outlined in the preceding section, currently available in vitro cell culture (e.g., cell suspensions, protoplasts), and organ and tissue culture techniques (differentiated tissues, callus, shoots, zygotic embryos, adventitious root, hairy roots) offer a wide range of procedures enabling the acquisition of therapeutically relevant molecules through tailored strategies that maximize their production [73]. Among the frequently employed methods, callus and suspension cultures derived from explants can be stimulated through media optimisation and elicitation to produce considerable amounts of secondary metabolites. For example, hairy root cultures induced by *Agrobacterium rhizogenes* combine rapid growth with genetic stability for efficient synthesis of root-specific compounds. Notably, to date, researchers have explored hairy root systems in over 100 species of medicinal plants [18]. External factors such as biotic or abiotic elicitors activate defence-related pathways that upregulate biosynthetic genes, thereby further increasing metabolite accumulation, and directing flux through key biochemical routes. In parallel, precursor feeding strategies enable a more direct modulation of metabolic flux by supplying key intermediates [74]. In this context, renewed attention has been given to precursor-based intervention strategies; the comprehensive review by Ramadani and Jadid [69] details how precursor supply, when timed and supplied correctly in organ, tissue or cell cultures, can significantly redirect metabolic flux toward high-value secondary metabolites.

Plant tissue culture therefore provides not only a production platform but also a controllable experimental system for evaluating and optimising different strategies aimed at enhancing specialized metabolite biosynthesis. At the genomic level, polyploidization, or whole-genome duplication, appears to be a sustainable and reproducible strategy for boosting the production of pharmaceutically relevant compounds in medicinal plants. It is usually induced by the application of antimitotic agents, most commonly colchicine or oryzalin. Increasing ploidy results in higher gene copy numbers and often leads to altered regulation of biosynthetic pathways, which can result in elevated levels of flavonoids, terpenoids, alkaloids, and other bioactive compounds [75,76]. The aforementioned approaches have been extensively documented in the context of plant tissue culture as a platform for optimising therapeutic secondary metabolite biosynthesis across diverse species and culture types. Notable recent reviews and original studies [75,76,77,78,79,80] provide comprehensive coverage of these strategies. Finally, integration of optimised culture into bioreactor setups enables scalable, standardized production. In parallel, advanced approaches, including metabolic engineering and selection of high-yielding cell lines, further refine biosynthetic outputs for industrial and therapeutic applications. Additionally, bioinformatic analysis may complement this by providing the massive amount of processed genomic and transcriptomic data gathered from computer modelling/simulation and a large volume of previous experiments [81]. Although this section focuses on plant tissue culture as the principal experimental platform, its comparative evaluation is intentionally presented together with those of the remaining biotechnological strategies discussed throughout this review. Accordingly, Table 2 provides a comparative synthesis of the principal advantages, limitations, scalability, regulatory considerations, and representative applications of plant tissue culture alongside the other major approaches.

**Table 2:** Comparative summary of biotechnological strategies.

Strategy	Main Advantages	Current Limitations/Challenges	Scalability	Typical Complementary Approaches	References
Plant tissue culture	Controlled, year-round production independent of seasonal and geographical constraints; reduced dependence on wild resources; reproducible cultivation conditions; rapid propagation and conservation of valuable genotypes; availability of diverse culture platforms; provides a controllable basis for further biotechnological interventions	Productivity remains strongly species-, genotype-, tissue- and culture-system-dependent; dedifferentiated cultures may lose tissue-specific biosynthetic capacity; prolonged cultivation may lead to genetic or epigenetic instability and declining productivity; biomass accumulation does not necessarily correlate with metabolite yield; contamination control, product recovery, long-term stability and scale-up remain challenging	Moderate to high for selected metabolites and culture systems, but not universally transferable. Suspension-cell, adventitious-root, and hairy-root cultures can be transferred to bioreactors, and large-scale cultivation has been demonstrated for selected systems. Broader implementation remains limited by shear sensitivity, aggregation, oxygen and nutrient transfer, culture instability, batch variability, product recovery, and production economics	Selection of high-producing lines; elicitation; precursor feeding; polyploidization; metabolic and genome engineering; omics-guided optimisation; bioreactor and process engineering	[73,82]
Elicitation	Rapid enhancement of specialized metabolite accumulation through endogenous defence and stress-signalling pathways; does not require stable genetic modification; applicable to diverse tissue, organ and cell culture systems; broad and expanding range of biotic, abiotic and physicochemical elicitors; emerging nanoelicitors further extend the range of available stress-based interventions	Responses are highly dependent on species, genotype, tissue or culture type, physiological state, elicitor identity, dose, exposure time and application stage; effective concentrations may approach levels causing oxidative damage, growth inhibition or biomass reduction; responses may be transient and difficult to reproduce during scale-up; increased metabolite concentration does not necessarily increase total yield; mechanisms and long-term consequences of many emerging elicitors remain insufficiently characterized	Moderate, primarily as a process-intensification strategy applied to an established culture platform. Elicitors can generally be introduced without major modification of the production system; however, reproducible scale-up is constrained by strong dependence on species, genotype, tissue or culture type, physiological state, dose, exposure time, and application stage. Transient responses and trade-offs between biomass accumulation and metabolite concentration may reduce process predictability	Precursor feeding; combined or sequential elicitation; metabolomics and transcriptomics; transcription-factor and pathway engineering; process optimisation; nanoelicitation as an emerging subclass	[15,17]
Precursor feeding	Directly increases the availability of biosynthetic intermediates and can redirect metabolic flux toward a target compound; relatively simple to apply in established in vitro systems; particularly useful when the biosynthetic pathway and limiting substrates are known; can be combined with elicitation to improve pathway activation and substrate availability simultaneously	Effectiveness depends on precursor identity, concentration, uptake, transport and conversion efficiency; incomplete pathway knowledge limits rational precursor selection; metabolic competition and feedback regulation may restrict conversion into the desired product; excessive concentrations may cause cytotoxicity or metabolic imbalance; production stability and economic feasibility depend on precursor cost and culture performance	Low to moderate and strongly dependent on precursor and process economics. Addition to an established in vitro system is technically straightforward, but scale-up requires consistent precursor uptake and conversion, controlled dosing, production stability, and avoidance of cytotoxicity or metabolic imbalance. Commercial feasibility may be limited by precursor cost and incomplete conversion into the target product	Elicitation; metabolomics and flux analysis; transcriptomics; transporter analysis; pathway engineering; optimisation of culture conditions and application timing	[69,82]
Polyploidization	Increased chromosome and gene copy number may alter pathway regulation and enhance the accumulation of selected specialized metabolites; may generate new chemotypes and increase biomass, organ size or stress tolerance in some species; once obtained and stabilized, polyploid lines can provide a reproducible source of improved material	Effects are highly species-, genotype-, tissue- and metabolite-dependent; increased ploidy may enhance, reduce or leave metabolite production unchanged; antimitotic treatments require optimisation and may reduce survival or regeneration; polyploids may display altered growth, fertility, morphology and developmental stability; genome-wide transcriptional and epigenetic consequences make metabolic outcomes difficult to predict	Low to moderate as a direct production strategy; more readily applicable as a breeding and line-development approach. The induction procedure itself is technically accessible, but implementation requires genotype-specific optimisation, regeneration and selection of stable polyploids, verification of ploidy, and confirmation that altered metabolite accumulation persists during propagation or cultivation. Metabolic outcomes are not uniformly positive and may include increased, reduced, or silenced pathway activity	In vitro regeneration and selection; flow-cytometric and cytological verification; metabolomics and transcriptomics; chemotype screening; computational or machine-learning-assisted optimisation of induction conditions	[83,84]
Metabolic engineering and genome editing	Enables targeted manipulation of biosynthetic enzymes, competing branches, transport processes, and transcriptional regulators; supports overexpression, silencing, knockout, and multiplex modification of pathway components; CRISPR/Cas systems provide comparatively precise and programmable editing and can facilitate functional validation of genes involved in specialized metabolism; modifications may provide stable improvements once successfully regenerated	Effective intervention requires sufficiently characterized pathways and reliable genomic and transcriptomic resources; pathway branching, feedback regulation, and interactions with primary metabolism may cause pleiotropic or compensatory responses; transformation and plant-regeneration efficiency remain major species-dependent bottlenecks; delivery of editing reagents, off-target changes, mosaicism and recovery of stable edited lines remain challenging; practical use is also shaped by biosafety and regulatory requirements	Moderate, with high long-term potential but substantial species- and platform-specific barriers. Scalability in medicinal plants and plant cultures depends on efficient transformation and regeneration, stable multigene expression, pathway knowledge, control of competing fluxes, product transport, and avoidance of cellular toxicity or feedback inhibition. Translation to commercial production also requires stable performance across generations or culture cycles and compliance with applicable regulatory frameworks	Tissue culture and regeneration; transcriptomics, metabolomics and flux analysis; bioinformatic target prediction; transcription-factor and enzyme engineering; synthetic biology; heterologous pathway reconstruction	[74,85]
Omics-guided approaches and bioinformatics	Genomics, transcriptomics, proteomics, and metabolomics enable identification of candidate enzymes, transporters and transcriptional regulators; comparative and integrated analyses support pathway reconstruction, tissue- and stage-specific characterization, gene mining and recognition of potential regulatory bottlenecks; bioinformatics can prioritize targets for functional validation, genome editing and metabolic engineering, it can shift optimisation from largely empirical screening toward knowledge-guided intervention	These approaches do not directly increase metabolite production without experimental validation and downstream engineering; medicinal-plant genomes and pathway annotations remain incomplete for many species; large and heterogeneous datasets require standardization, integration and substantial computational expertise; specialized metabolism is dynamic, tissue-specific and environmentally responsive, complicating causal interpretation; predictions may generate numerous candidates but cannot by themselves establish enzymatic function or pathway control	High analytical scalability but indirect production scalability. Once standardized datasets and computational workflows are available, large numbers of genes, pathways, metabolites, and regulatory candidates can be analysed in parallel. Their contribution to production nevertheless depends on experimental validation and subsequent implementation through culture optimisation, breeding, metabolic engineering, or genome editing; heterogeneous datasets, limited standardization, computational expertise, and validation requirements remain important bottlenecks	Functional genomics; gene-expression and enzyme validation; metabolic and genome engineering; metabolomics-guided elicitation and culture optimisation; metabolic-flux modelling; machine learning and network analysis	[86,87]

Abbreviations: CRISPR, Clustered Regularly Interspaced Short Palindromic Repeats; Cas, CRISPR-associated protein.

Among the complementary interventions applicable within plant tissue culture systems, elicitation has received considerable attention in the literature. It represents one of the most effective approaches for enhancing specialized metabolite production in plant tissue cultures. Elicitors are biotic or abiotic signalling factors that trigger biochemical and physiological responses, leading to activation of plant defence mechanisms and subsequent induction of secondary metabolic pathways. Rather than directly targeting secondary metabolite biosynthesis, elicitation deliberately perturbs cellular homeostasis, thereby activating endogenous defence responses, with enhanced metabolite accumulation representing a downstream consequence of this adaptive process [88]. Numerous studies have demonstrated that controlled induction of stress responses can significantly increase the accumulation of pharmacologically relevant compounds in vitro systems. For example, elicitation, involving the exposure cells to abiotic or biotic stimuli such as methyl jasmonate, salicylic acid, or specific light, salt or UV stressors can activate defence-linked biosynthetic pathways, leading to their elevated accumulation [15,89]. In contrast, relatively fewer reports have investigated the potential of abiotic nanoparticle-based elicitors (nanoelicitors) as modulators of secondary metabolism. Given the emerging interest in this area, the following section focuses specifically on the role of nanoparticle-based elicitors in enhancing the production of therapeutically relevant metabolites.

Nanotechnology has become an important component of contemporary biotechnology, with nanoparticles (NPs) representing a particularly interesting class of agents used in plant tissue culture systems. In plant tissue cultures, nanoparticles have been investigated for their potential to control microbial contamination, improve nutrient availability, stimulate plant growth, and act as elicitors under carefully optimised experimental conditions. However, these responses are highly dependent on nanoparticle concentration, composition, plant species, tissue type, and experimental conditions. Non-optimised treatments may induce phytotoxic responses rather than beneficial metabolic effects [90]. Current evidence for nanoelicitation remains largely preliminary and is derived predominantly from controlled laboratory studies. Consequently, further investigations are required before these approaches can be considered broadly applicable in plant biotechnology.

Various types of nanoparticles, including Au, Ag, Ag_2_O, TiO_2_, ZnO, CuO, and Fe_3_O_4_, have been reported as effective unconventional elicitors stimulating secondary metabolite biosynthesis [91,92,93]. A possible mechanism by which nanomaterials act as elicitors is their interaction with plant genomes leading to increased expression of genes involved in the biosynthesis of active metabolites [88,94]. A parallel indirect mechanism involves the above-mentioned overproduction of ROS in response to CNP application. ROS can subsequently modulate enzyme activities and the expression of genes associated with plant stress adaptation, including those involved in the glutathione–ascorbate cycle, the shikimate pathway, and the phenylpropanoid pathway, from which a large proportion of plant bioactive compounds are derived [95]. Among the wide range of nanomaterials investigated, carbon nanoparticles have attracted particular attention because of their distinctive physicochemical properties and their interactions with plant stress responses.

**Figure 2:**
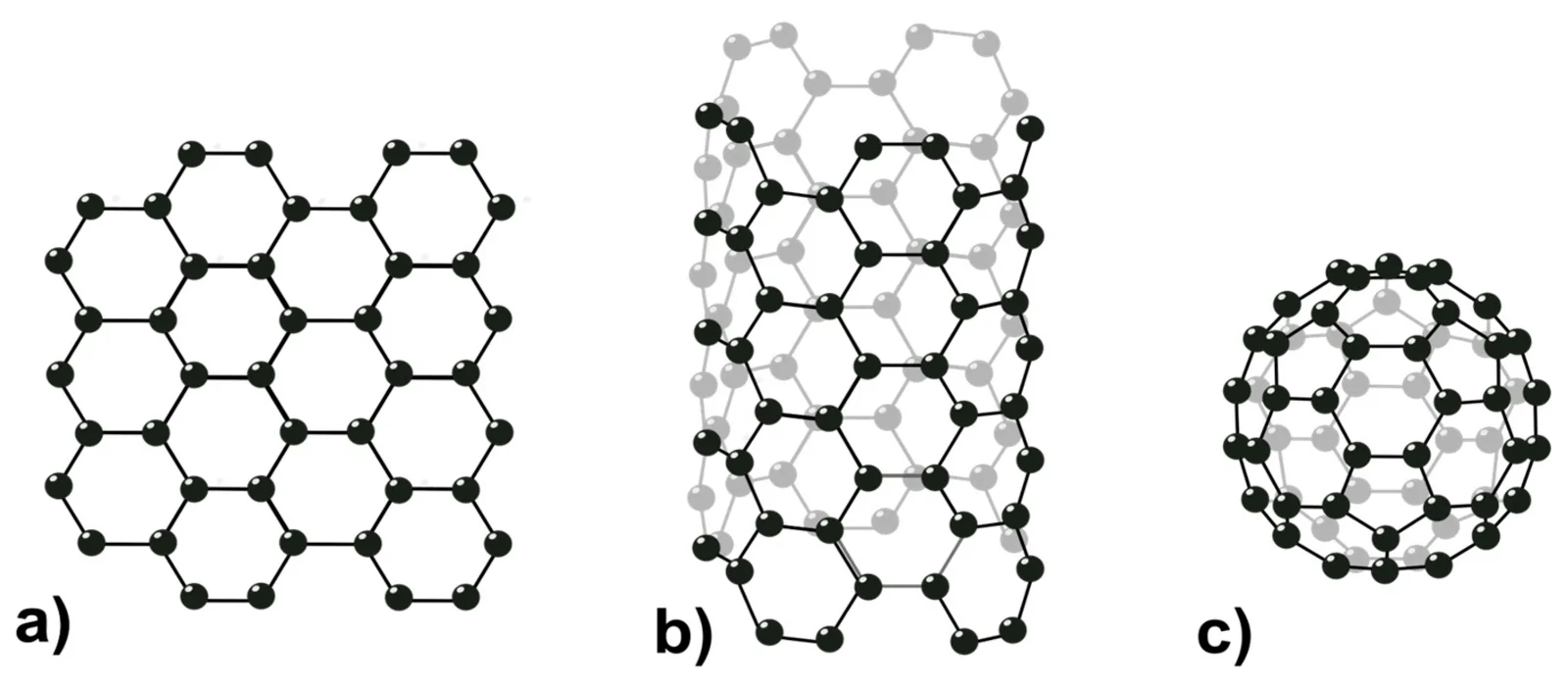
Examples of nanoscale materials based on carbon atoms: (**a**) graphene, (**b**) nanotube, (**c**) fullerene.

**Figure 3:**
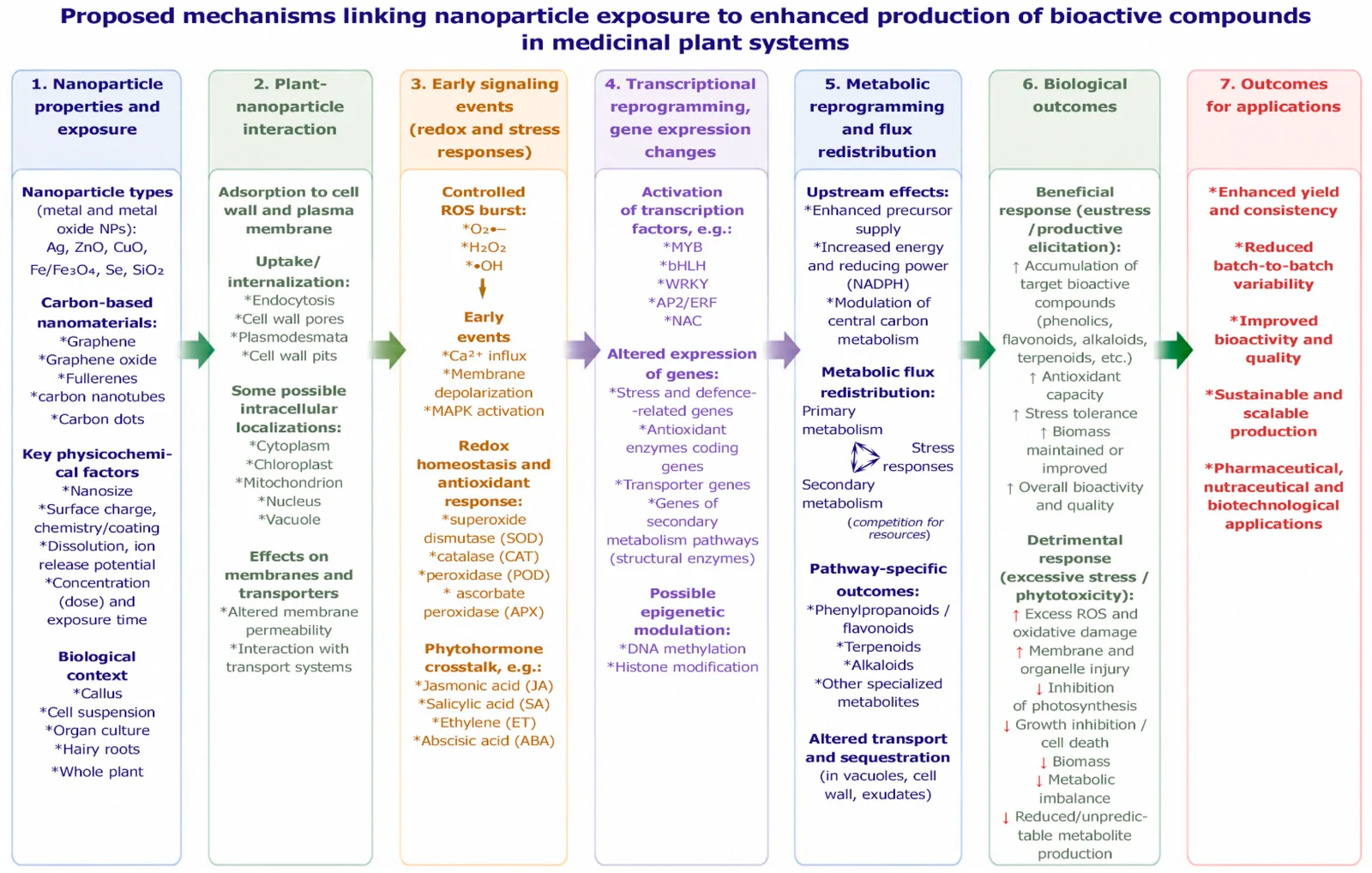
Mechanisms Linking Nanoparticle Exposure to Bioactive Compound Production. Nanoparticle-induced responses are influenced by the physicochemical properties of the nanomaterials (e.g., composition, size, surface characteristics, concentration, and exposure duration) as well as by the biological context, including plant species, tissue type, and in vitro culture system. Following interaction with plant cells, nanoparticles may trigger transient reactive oxygen species (ROS) generation and stress-related signalling pathways, leading to transcriptional reprogramming, altered expression of biosynthetic and regulatory genes, and redistribution of metabolic flux. Depending on the intensity of the response, these processes may promote the accumulation of therapeutically relevant secondary metabolites (productive elicitation) or, under excessive stress conditions, result in phytotoxic effects, growth inhibition, and metabolic imbalance. The proposed sequence represents a conceptual synthesis of the current evidence and highlights the context-dependent nature of nanoparticle-mediated modulation of secondary metabolism. Abbreviations: ABA, abscisic acid; AP2/ERF, APETALA2/ethylene response factor; APX, ascorbate peroxidase; bHLH, basic helix-loop-helix transcription factor; CAT, catalase; ET, ethylene; H_2_O_2_, hydrogen peroxide; JA, jasmonic acid; MAPK, mitogen-activated protein kinase; MYB, MYB transcription factor; NAC, NAC transcription factor; NADPH, reduced nicotinamide adenine dinucleotide phosphate; O_2_•^−^, superoxide anion; •OH, hydroxyl radical; POD, peroxidase; ROS, reactive oxygen species; SA, salicylic acid; SOD, superoxide dismutase; WRKY, WRKY transcription factor.

Carbon nanoparticles (CNPs) are nanoscale materials (1–100 nm) composed primarily of carbon atoms. The most extensively investigated classes include carbon nanotubes (CNTs), fullerenes, graphene oxide (GO) (Figure 2), and carbon quantum dots (CQDs) [96,97]. These materials possess distinctive physicochemical properties, including high specific surface area, electrical conductivity, redox activity, and tunable surface chemistry, which underlie their interactions with biological systems [98]. Among carbon-based nanomaterials, fullerene derivatives appear particularly relevant as nanoelicitors because their cage-like architecture and redox-modulating properties enable interactions with plant stress-responsive regulatory pathways involved in specialized metabolite biosynthesis. Accumulating evidence further suggests that fullerenes influence oxidative homeostasis, cellular signalling, and downstream metabolic responses associated with plant adaptation and secondary metabolism [95,99,100]. A conceptual synthesis of the current evidence regarding the proposed mechanisms linking nanoparticle exposure with modulation of secondary metabolism in medicinal plant systems is presented in Figure 3.

Due to their increasing use, CNPs may enter soil and aquatic environments, where they interact with plants. These interactions can produce either stimulatory or toxic effects, depending on nanoparticle properties and exposure conditions. This duality is particularly relevant for nanoelicitation. The stress responses that stimulate specialized metabolite biosynthesis may also compromise plant physiology when nanoparticle exposure exceeds optimal conditions. Empirical evidence indicates that nanoparticles may enter the environment through domestic and industrial wastewater, sewage sludge, industrial emissions, landfills, and surface run-off [101,102,103,104]. They may also be taken up by plants and subsequently enter the food chain [105,106]. Consequently, nanomaterials are increasingly recognised as emerging environmental contaminants, although harmonized regulatory standards and exposure thresholds remain limited [107]. These uncertainties highlight the need for standardized analytical methods, long-term environmental monitoring, and studies addressing nanoparticle transformation, environmental persistence, and food-chain transfer [108,109,110]. The application of nanoelicitors is also associated with potential phytotoxic effects. Depending on nanoparticle type and exposure conditions, these may include reductions in chlorophyll content, cell viability, photosynthetic efficiency, and sugar accumulation, as well as inhibition of seed germination [94,111]. Phytotoxic effects have also been reported for fullerenes (nC60), including obstruction of root-cell pores, reduced transpiration, and disruption of cell membrane fluidity [112]. At present, evidence regarding the effects of CNPs on specialized metabolite production remains limited and is derived predominantly from controlled laboratory studies. Consequently, the available findings should be interpreted cautiously and cannot yet be generalized across nanoparticle types, plant species, tissue types, or experimental conditions. Representative examples of CNP-induced modulation of specialized metabolite accumulation are summarized in Table 3.

Collectively, these observations illustrate that individual biotechnological interventions cannot be evaluated independently of the biological context in which they are applied. Plant tissue culture itself alters physiological, metabolic, genetic, and epigenetic states, thereby influencing subsequent responses to elicitation, metabolic engineering, or genome editing. Consequently, combining multiple approaches should not be regarded as a simple additive process. Future progress will depend not only on integrating different technologies but also on understanding their interactions across multiple regulatory levels to develop predictive rather than empirical optimisation strategies.

## 4. Engineering and Omics Technologies in Plant Compound Production

Alongside in vitro culture-based biotechnology, increasingly efficient synthesis of therapeutic compounds is achieved through engineering-driven strategies. These approaches, however, require molecular information, for example, the genomes, proteomes, metabolic pathways or regulatory networks of medicinal plants. Modern strategies aimed at engineering and modifying metabolic pathways in medicinal plants increasingly rely on large-scale molecular datasets generated across multiple analytical layers. The interpretation of such bulk information requires systematic integration to formulate biologically meaningful hypotheses, resolve regulatory relationships, and guide targeted interventions in secondary metabolism. Consequently, the coordinated use of genomics, transcriptomics, proteomics, metabolomics, and related approaches has become an operational necessity rather than an auxiliary tool. Accordingly, contemporary metabolic engineering increasingly shifts from single-gene interventions toward multi-layered strategies in which omics-derived knowledge, computational modelling, and molecular engineering are integrated to enable more predictable control of metabolic networks. This integrative, systems-oriented framework has been collectively conceptualized as herbgenomics, reflecting a data-driven strategy for deciphering biosynthetic networks, regulatory hierarchies, and molecular mechanisms underlying the production and activity of medicinal plant-derived bioactive compounds [118]. Within this context, herbgenomics supports rational metabolic engineering, functional modification of biosynthetic pathways, and the sustainable optimisation of therapeutically relevant metabolites, including those traditionally exploited in systems such as Traditional Chinese Medicine (TCM) [119,120]. On this basis, several core intervention strategies have been developed at the conceptual level: enhancing flux through rate-limiting steps, redirecting metabolism away from competing pathways, and modifying enzyme properties to improve catalytic performance. These strategies are implemented using a range of molecular techniques, including overexpression of endogenous or enzymes, manipulation of transcription factors, targeted gene silencing, genome editing, and coordinated co-overexpression of multiple pathway components. Together, these approaches enable both direct modulation of enzymatic activity and higher-level control of pathway regulation, providing flexible tools for increasing the yield of therapeutically valuable metabolites [121]. Building on this regulatory architecture, metabolic engineering has emerged as a strategy to deliberately reprogram metabolic capabilities through targeted genetic manipulation. Initially developed in microbial systems using recombinant DNA and pathway engineering approaches, metabolic engineering enables the redirection and intensification of metabolic fluxes toward desired products within relatively short timeframes. It should be noted that, for some medicinal plants, engineering of native biosynthetic pathways remains constrained by incomplete pathway elucidation, limited transformation efficiency, or poor regeneration capacity. In such cases, heterologous production platforms provide a complementary approach for reconstructing and evaluating selected biosynthetic pathways.

The effective deployment of these engineering strategies critically depends on access to high-quality genomic information. In this context, advances in medicinal plant genome sequencing have provided the necessary molecular foundation for precise pathway reconstruction and regulatory manipulation. Since the Sanger-based sequencing of the first medicinal plant nuclear genome, *Ricinus communis*, in 2010 [122], plant genomics has undergone rapid methodological and technological advancement. Early medicinal plant genome projects were constrained by short-read sequencing. This constraint was addressed by third-generation sequencing platforms such as PacBio and Oxford Nanopore, which generate long reads spanning several to tens of kilobases and substantially improve genome assembly quality. Although these technologies were introduced around 2010, their routine application in medicinal plant genomics accelerated only after 2016. Since then, improved accessibility and reduced sequencing costs have driven a rapid increase in high-quality medicinal plant genome assemblies, particularly from 2020 onward, resulting in greater assembly continuity, accuracy, and reduced structural errors [120]. Large-scale genome sequencing initiatives, including the 10KP, the 1K Medicinal Plant Genome Project, and the Earth Biogenome Project, have markedly expanded genomic resources for medicinal plants. By November 2024, the 1K Medicinal Plant Genome Database listed 107 sequenced medicinal plant genomes, with additional genomes reported beyond this collection [123,124].

Comprehensive knowledge of medicinal plant genome sequences provides a predictive framework for further analysis. Genome-scale sequence information enables reliable prediction of coding potential, regulatory elements, and transcription factor repertoires; consequently, it strengthens the ability to engineer transcriptional regulation of specialized metabolic pathways and to enhance the accumulation of bioactive compounds. Within this framework, plant secondary metabolite biosynthesis emerges as a highly coordinated process governed by tight, multi-level regulation. At its core, this regulation relies on transcription factors acting as central nodes that coordinate the expression of enzyme-encoding genes across entire biosynthetic pathways. In their natural context, plants deploy complex transcriptional networks as part of sophisticated defense mechanisms against biotic and abiotic stresses. Transcription factors from multiple families (at least 58 known) integrate developmental cues with environmental signals, thereby modulating pathway gene expression and controlling secondary metabolite accumulation [125,126]. As a result, biosynthetic output reflects the combined action of numerous transcriptional regulators operating at distinct regulatory levels. To maintain proportional and transient metabolic responses, transcription factors are themselves subject to stringent regulatory control, ensuring homeostasis of regulatory activity within the cell. Beyond systemic feedback operating at the pathway level, regulatory mechanisms frequently act directly on the transcription factor to modulate its abundance and activity. Two principal modes contribute to this control. Transcriptional autoregulation relies on feedback loops in which a transcription factor modulates its own gene expression, often in a negative manner, thereby preventing sustained or excessive activation. Within the AP2/ERF superfamily, AtERF1 in *Arabidopsis thaliana* illustrates this mode by undergoing stress-induced activation followed by repression of its own transcription, which attenuates defence-associated metabolite accumulation once the stimulus declines. Post-translational regulation provides an additional layer of control by rapidly adjusting transcription factor stability, activity, or subcellular localization. This mode is reflected in AP2/ERF factors such as NtERF172 in *Nicotiana tabacum*, whose phosphorylation-dependent degradation limits jasmonate-responsive metabolic activation and fine-tunes regulatory output in accordance with stress intensity and duration [127,128]. Taken together, these observations underscore that secondary metabolite production is not only regulated at the level of biosynthetic genes but also through multilayered control of transcription factors themselves. The existence of dual regulatory modes acting on transcription factors, combined with the presence of higher-order master regulators (e.g., *A. thaliana* glucose-modulated master regulators, HXK1, KIN10/11, TOR) that orchestrate entire transcriptional programs, highlights the conceptual and technical demands associated with rational pathway manipulation [129]. At the same time, this hierarchical organization provides multiple leverage points for targeted intervention, substantially expanding the scope of strategies available for fine-tuning specialized metabolism.

At the mechanistic level, transcription factors influence secondary metabolite biosynthesis through both direct and indirect regulatory mechanisms. In direct regulation, factors such as members of the AP2/ERF family bind specific *cis*-regulatory elements within promoters of biosynthetic genes, leading to rapid activation or repression of pathway enzymes. This mode enables precise and timely adjustment of metabolic output in response to developmental or environmental signals. In contrast, indirect regulation operates through transcriptional cascades or signalling hubs, in which transcription factors act upstream in regulatory networks and modulate secondary metabolism without directly interacting with terminal biosynthetic gene promoters. Together, these complementary mechanisms provide flexibility and robustness to transcriptional control of specialized metabolic pathways [128].

The body of literature addressing the engineering of biosynthetic regulatory mechanisms to enhance the accumulation of therapeutically relevant plant secondary metabolites has expanded substantially in recent years. Members of major transcription factor families, including WRKY, bHLH, MYB, NAC, AP2/ERF and bZIP, have been repeatedly implicated in the regulation of pathways leading to the production of alkaloids, terpenoids, flavonoids, and phenylpropanoids [130,131,132]. For example, Jia et al. [131] reported that MYB-type transcription factors can enhance the accumulation of either tanshinones or phenolic acids. They are the major therapeutic constituents of *Salvia miltiorrhiza*, widely used in cardiovascular disease treatment. However, their regulatory effects remain constrained by pathway specificity and intrinsic network antagonism. To overcome these limitations, an artificially engineered transcription factor, SmMYB36-VP16, was developed to reprogram transcriptional control at a higher regulatory level. The expression of this synthetic regulator enabled the concurrent enhancement of both metabolite classes compared with wild-type tissues, demonstrating that artificial transcription factor engineering, representing a synthetic biology-oriented strategy, can bypass native regulatory constraints and effectively optimise medicinal metabolite production [131].

Even with genetic modifications at the regulatory level, achieving a balanced metabolic flux remains a central challenge in plant metabolic engineering. Efficient allocation of precursor pools, cofactors, and cellular energy toward target compound biosynthesis is essential to maximize product yield without compromising host viability. To address these constraints, enzyme-centered strategies have been widely employed, including enzyme engineering to enhance catalytic efficiency or alleviate substrate inhibition, subcellular targeting to co-localize pathway intermediates and enzymes, and modulation of competing pathways to minimize precursor diversion. Enzyme engineering, achieved through rational design or directed evolution, can improve turnover rates, substrate specificity, or stability under production-relevant conditions. Subcellular targeting, such as directing pathway enzymes to plastids or endoplasmic reticulum membranes, exploits organelle-specific metabolite pools and cofactor availability, thereby increasing conversion efficiency and overall pathway throughput. Once biosynthetic pathways have been elucidated at the enzymatic level, a widely applied strategy to enhance secondary metabolite production is the overexpression of endogenous, pathway-resident enzymes. In particular, increasing the abundance of rate-limiting enzymes can elevate precursor availability and drive metabolic flux toward downstream products. These enzymes catalyse diverse biochemical transformations, including methylation, condensation, isomerization, glycosylation, and acylation, and collectively define both the efficiency and directionality of specialized metabolism [121,133].

Compared with more disruptive interventions, the manipulation of native enzymes, through overexpression or targeted silencing, often operates within pre-existing regulatory architectures, rendering feedback responses more predictable and physiologically tolerable. However, the effectiveness of endogenous enzyme overexpression is strongly influenced by pathway complexity and feedback regulation. Specialized metabolic pathways typically consist of numerous interconnected enzymatic steps and are embedded within multilayered regulatory networks that integrate developmental programs and environmental cues. As a consequence, isolated overexpression of single enzymes does not always yield proportional increases in product accumulation, owing to feedback inhibition, intermediate toxicity, or downstream bottlenecks. These constraints underscore that successful endogenous enzyme engineering generally requires coordinated modulation of multiple pathway components to preserve metabolic balance and sustain flux [134].

An additional and complementary strategy involves expressing heterologous genes encoding biosynthetic enzymes from other plant species in the target medicinal plant, thereby complementing its endogenous metabolic machinery. The introduction of key rate-limiting enzymes with superior catalytic properties can enhance the efficiency of specific metabolic conversions within endogenous pathways. Such enzymes may display higher substrate affinity, improved kinetic parameters, or reduced sensitivity to feedback inhibition relative to their native counterparts, thereby enabling more effective redirection of metabolic flux toward desired end products. When integrated with pathway balancing and computational metabolic modelling, heterologous enzyme expression can substantially increase yields while limiting the accumulation of unwanted intermediates or side products. A representative example is the enhancement of scopolamine production, a tropane alkaloid of high therapeutic relevance owing to its anticholinergic activity and clinical use in the treatment of motion sickness and postoperative nausea. This was achieved through heterologous expression of rate-limiting enzymes, illustrating the translational potential of enzyme-centered metabolic engineering [121,133,135].

Beyond transcriptional and enzyme-level interventions, genome editing has emerged as a complementary strategy for precise, heritable reprogramming of metabolic pathways in medicinal plants. Advances in genetic engineering, particularly the emergence of CRISPR/Cas9 (considered a third generation of gene-editing technologies relative to ZFN and TALEN), have fundamentally transformed the targeted manipulation of biosynthetic pathways in medicinal plants. Unlike earlier protein-based nucleases, CRISPR/Cas9 enables precise and efficient modification of both biosynthetic genes and regulatory elements, facilitating the enhancement of enzyme activity at rate-limiting steps, suppression of competing pathways consuming shared precursors, and removal of feedback inhibition constraints. Through such interventions, metabolic flux can be effectively redirected toward desired end products, resulting in substantial yield improvements of specialized metabolites, including terpenoids. Despite these advantages, genome editing in medicinal plants remains constrained by species-specific regeneration barriers, variable transformation efficiencies, and reliance on genotype-dependent tissue culture systems. Consequently, current efforts increasingly focus on next-generation delivery and regeneration platforms, such as protoplast-based editing and tissue culture-independent transformation approaches [136,137]. An application of CRISPR/Cas9-mediated metabolic editing has been demonstrated in *Symphytum officinale*. It is a medicinal plant valued for its anti-inflammatory and analgesic properties but limited by the presence of hepatotoxic pyrrolizidine alkaloids. Using a targeted genome-editing approach, mutations were introduced into the gene encoding homospermidine synthase, a key enzyme responsible for the formation of homospermidine, the committed precursor of pyrrolizidine alkaloid biosynthesis. Disruption of this gene resulted in a complete elimination of pyrrolizidine alkaloids in edited hairy root cultures, while preserving the medicinally relevant metabolic background [138]. Recent advances and remaining challenges in medicinal plant genome editing have been comprehensively reviewed by Chen et al. [137], highlighting genome editing as a central and independent domain of contemporary medicinal plant biotechnology.

In parallel to genome editing approaches, gene silencing constitutes another strategy in metabolic engineering of medicinal plants. Rather than introducing permanent modifications at the DNA level, gene silencing enables the targeted reduction of gene expression, providing a means to suppress undesirable metabolic activities or to enhance the accumulation of specific metabolites by attenuating competing pathways. This regulatory strategy has been successfully employed to modulate secondary metabolism, particularly in pathways where complete gene disruption would compromise cellular viability or developmental integrity. Gene silencing is predominantly achieved through RNA-based mechanisms, including RNA interference, microRNA-mediated regulation, and virus-induced gene silencing. These approaches operate at the post-transcriptional level, resulting in substantial transcript depletion (knockdown) without fully abolishing gene function. As a consequence, gene silencing allows graded and reversible control over metabolic flux, offering a level of regulatory flexibility that is difficult to achieve through permanent genome edits alone. This feature is especially advantageous in complex biosynthetic networks characterized by extensive feedback regulation and pathway interdependence [139,140].

Conceptually, gene silencing complements genome editing by expanding the regulatory space available for metabolic optimisation: while genome editing enables stable pathway restructuring, gene silencing provides dynamic fine-tuning of pathway activity. Together, these strategies underpin modern metabolic engineering frameworks and form a logical bridge toward plant cell culture engineering, where cellular systems are deliberately designed and conditioned for optimised metabolite production. In such systems, genetic interventions, whether permanent or reversible, are integrated with controlled culture conditions to shape biosynthetic capacity in a predictable and scalable manner.

## 5. Bioinformatics for Boosting Synthesis of Bioactive Metabolites

The integration of bioinformatics with high-throughput omics technologies has become an essential pillar in modern natural product research. Bioinformatics provides computational frameworks for the systematic exploration of large-scale molecular datasets, enabling the identification and prioritization of candidate bioactive compounds in medicinal plants. This approach enhances the efficiency of bioprospecting by allowing researchers to predict the likelihood of discovering therapeutically relevant metabolites in related species. In consequence, it reduces reliance on random screening methods. Computational analyses facilitate early-stage compound annotation, structural characterization, and assessment of physicochemical properties, molecular interactions, and predicted biological activity, guiding the selection of promising candidates for further experimental validation. In the context of the advent of multi-omics technologies, the use of advanced data-driven tools has become indispensable, as these platforms generate extensive datasets requiring integrative analyses. Transcriptomic analyses, for instance, allow the identification of candidate biosynthetic genes, transcription factors, and regulatory elements associated with metabolite accumulation. This approach is especially valuable when complete genome sequences are unavailable for many medicinal plant species. In such cases, transcriptome-based analyses provide a powerful alternative for gene discovery and pathway reconstruction. When integrated with metabolomic profiling, these data enable correlation-based approaches linking gene expression patterns to metabolite abundance, thereby revealing functional insights into specialized biosynthetic pathways [141].

In drug development, these analyses are supported by a wide range of curated databases and bioinformatic resources. In 2020, Sorokina and Steinbeck [142] reported a rapid expansion of natural product databases and collections over the past two decades. Since 2000, more than 120 resources have been published and reused, with 98 still partially accessible and 50 freely available as open-access platforms. This growth reflects both general-purpose and thematic repositories, highlighting the increasing volume and diversity of data supporting natural product research [142]. Some of them are specifically directed toward targeted therapeutic interventions. Generally, sequence storage and annotation commonly rely on the National Center for Biotechnology Information (NCBI) (https://www.ncbi.nlm.nih.gov), non-redundant and nucleotide databases, as well as on European Bioinformatics Institute of the European Molecular Biology Laboratory (EMBL-EBI) (https://www.embl.org), and DNA Data Bank of Japan (DDBJ) (https://www.ddbj.nig.ac.jp) resources. Together, they create The International Nucleotide Sequence Database Collaboration (INSDC) (https://www.insdc.org/) and exchange data with each other. In parallel, there are several protein sequence databases, including UniProtKB/Swiss-Prot (https://www.expasy.org/resources/uniprotkb-swiss-prot, accessed on 1 February 2026), wwPDB (https://www.wwpdb.org/, accessed on 1 February 2026)) or World-2DPAGE (https://bio.tools/world-2dpage, accessed on 1 February 2026)) [143,144]. These and other repositories (e.g., gene expression and biomarker databases) were thoughtfully presented in an excellent review by [19]. Pathway-level interpretation is frequently conducted using the Kyoto Encyclopedia of Genes and Genomes (KEGG) (https://www.genome.jp/kegg, accessed on 1 February 2026)), which maps genes and metabolites onto conserved biosynthetic routes. KEGG Drug additionally provides data on natural product–target interactions, linking plant metabolism to pharmacological research. Complementary platforms such as PathPred (https://www.genome.jp/tools/pathpred, accessed on 1 February 2026)) allow prediction of multistep biosynthetic reactions for structurally related metabolites, aiding pathway reconstruction in species lacking complete genome sequences. Public repositories such as the Gene Expression Omnibus (GEO; https://www.ncbi.nlm.nih.gov/geo, accessed on 1 February 2026)) and the EMBL-EBI ArrayExpress database (https://www.ebi.ac.uk/biostudies/arrayexpress, accessed on 1 February 2026)), together with plant-oriented databases such as PLEXdb (https://plexdb.org, accessed on 1 February 2026)), offer large-scale gene expression datasets suitable for co-expression network analysis. Network-based analyses, including tools such as WGCNA (Weighted Gene Co-expression Network Analysis) [145], facilitate identification of gene modules associated with specific metabolic traits or stress responses, supporting the rational selection of candidate genes for functional validation [19]. Beyond candidate identification, integrative network analyses increasingly enable reconstruction of system-level relationships linking genes, metabolites, and environmental variables. Such approaches shift medicinal plant research from descriptive pathway mapping toward predictive modelling of metabolic responses and biosynthetic outputs. In the context of medicinal plants, network-based pharmacology represents a highly valuable approach for elucidating bioactive interactions. It enables the prediction of bioactive compounds from complex mixtures of plant metabolites. It also provides an integrated view of their simultaneous interactions with diseases, which often involve multiple signalling pathways and biological targets [141,146].

The practical value of bioinformatic approaches lies in translating computational predictions into experimentally testable targets for metabolic engineering, genome editing, and other strategies aimed at optimising specialized metabolite production. Machine learning models integrating multiple omics layers can prioritize candidate genes potentially involved in terpenoid, alkaloid, and phenolic biosynthesis, thereby reducing the number of candidates requiring functional evaluation [147]. However, computational prediction alone does not establish enzymatic function or demonstrate increased production. A subsequent level of evidence is provided by integrated genomic, transcriptomic, and metabolomic studies, such as the identification and functional validation of an O-methyltransferase involved in polymethoxylated-flavonoid biosynthesis in *Citrus reticulata* cv. Chachiensis [148]. The direct production relevance of bioinformatics-supported modelling was demonstrated in *Nothapodytes nimmoniana*, where a genome-scale metabolic model prioritized strictosidine synthase as an engineering target and its experimental overexpression increased camptothecin accumulation approximately fivefold relative to the wild-type culture [149]. Together, these examples illustrate a progression from computational candidate prioritization, through functional validation, to model-guided metabolic intervention and measurable improvement in metabolite production.

Collectively, bioinformatics and multi-omics frameworks enable the identification, contextualization, and prioritization of bioactive compounds and therapeutic targets within complex biological systems, including medicinal plant metabolites. These insights establish a scalable platform for analysing and manipulating secondary metabolism. By uncovering key biosynthetic genes, regulatory networks, and pathway bottlenecks, such approaches accelerate the discovery of natural compounds and guide tissue culture and engineering strategies to enhance the production of therapeutically relevant metabolites.

## 6. Conclusions

The evidence discussed throughout this review indicates that isolated interventions rarely provide sufficient control over the highly interconnected networks governing specialized metabolism. Consequently, future progress in medicinal plant biotechnology will increasingly depend on the integration of complementary strategies operating across multiple biological levels rather than on further refinement of individual technologies alone. In particular, the growing availability of genomics, transcriptomics, proteomics, metabolomics, and computational approaches is transforming the field from empirical optimisation toward predictive, knowledge-driven engineering. Despite these advances, the development of robust, reproducible, and economically viable large-scale production platforms remains one of the principal challenges limiting broader industrial implementation. Finally, continued technological progress should be accompanied by careful consideration of biosafety, regulatory, and environmental aspects, particularly in relation to genome editing and nanoparticle-based approaches, to ensure their safe and sustainable application. At the same time, integrating multiple biotechnological approaches should not be regarded as a simple additive process. Future progress will depend on understanding and predicting interactions among regulatory processes operating across different biological levels.

## Figures and Tables

**Table 1:** Representative examples of the current biotechnological landscape of selected medicinal plants: species-specific strategies, opportunities, and limitations.

Species	Major Bioactive Compounds	Principal Therapeutic/Application Area	Representative Biotechnological Strategy	Main Advantages	Challenges and Limitations	References
*Artemisia annua* L.	Artemisinin; artemisinic acid	Antimalarial therapy (artemisinin-based combination therapies)	Predominant strategy: metabolic engineering of the native artemisinin pathway through coordinated regulation of biosynthetic enzymes, transcription factors (TF), and glandular trichome development.	Direct manipulation of pathway flux while preserving the native cellular context for efficient artemisinin accumulation	Pathway balance, trichome development and genotype-dependent responses limiting predictable yield improvement and large-scale production	[20,21]
*Camptotheca acuminata* Decne	Camptothecin	Anticancer (topoisomerase I inhibitor; source of irinotecan and topotecan derivatives)	In vitro culture optimisation, high-producing cell-line selection and elicitation, supported by omics-guided pathway discovery and targeted metabolic engineering	Controlled year-round production, systematic selection of high-producing lines and optimisation of elicitor responses	Camptothecin accumulation dependent on tissue type, culture line and elicitation conditions; incomplete elucidation of the downstream biosynthetic pathway limiting predictable metabolic engineering and large-scale production	[22,23]
*Cannabis sativa* L.	Cannabidiol (CBD); Δ9-tetrahydrocannabinol (THC); cannabigerol (CBG); terpenes	Pain/spasticity and selected neurological indications; extensive pharmacological research	In vitro propagation and regeneration systems, complemented by adventitious and hairy-root cultures for metabolic studies and specialized metabolite production	Controlled in vitro culture systems supporting propagation of elite genotypes, functional studies and specialized metabolite production	Genotype-dependent regeneration, limited transformation efficiency and incomplete whole-plant regeneration restricting broader application of advanced genetic engineering	[24,25]
*Catharanthus roseus* (L.) G. Don	Vinblastine; vincristine; vindoline; catharanthine	Anticancer therapy, particularly haematological malignancies	Pathway and transcription factor engineering in plant tissues and precursor-producing modules	More predictable control of metabolic flux through transcription factor-mediated regulation	Highly compartmentalised and multicellular pathway; technically complex vinblastine/vincristine production; limited production of downstream TIAs in cell and hairy-root cultures	[26]
*Curcuma* *longa* L.	Curcumin; demethoxycurcumin; bisdemethoxycurcumin	Anti-inflammatory products and extensive anticancer/ metabolic research	Hairy-root and microrhizome culture systems combined with elicitation for curcuminoid production	Genetically stable hairy-root cultures enabling sustained curcuminoid production and elicitor-based optimisation	Curcuminoid accumulation dependent on genotype, culture conditions and elicitation regime; further optimisation required for large-scale production and pathway engineering	[27,28]
*Digitalis lanata* Ehrh.	Digoxin, digitoxin, lanatoside C and related cardenolides	Cardiac glycosides used in selected heart-failure and arrhythmia settings	In vitro cultures based on precursor feeding, biotransformation and elicitation for enhanced cardenolide production	Reproducible cardenolide production, process optimisation and selective bioconversion of pharmaceutically important glycosides, including digoxin	Low and variable de novo cardenolide biosynthesis, dependence on culture conditions and precursor availability, and limited translation of emerging genetic approaches to large-scale production	[29,30]
*Echinacea purpurea* (L.) *Moench*	Cichoric acid; caftaric acid; chlorogenic acid; echinacoside (species-dependent)	Herbal products for upper respiratory tract symptoms and immunomodulatory research	Hairy-root culture combined with elicitation to enhance chicoric acid and other caffeic acid derivatives; priming as a complementary strategy	Genetically stable hairy-root cultures supporting sustained production of caffeic acid derivatives under controlled conditions	Species- and metabolite-dependent optimisation of elicitation protocols; challenging standardisation of metabolite profiles	[31,32]
*Ginkgo* *biloba* L.	Ginkgolides A, B and C; bilobalide; flavonol glycosides	Cognitive impairment, peripheral circulation and PAF antagonist research	In vitro culture systems combined with culture optimisation and elicitation for flavonoid and terpene trilactone production	Controlled production of bioactive metabolites independent of environmental variability and propagation of elite genotypes	Slow growth, genotype-dependent metabolite accumulation and incomplete understanding of metabolite regulation limiting large-scale production	[33,34]
*Glycyrrhiza glabra* L. */Glycyrrhiza* spp.	Glycyrrhizin (glycyrrhizic acid); liquiritin; isoliquiritigenin	Expectorant and gastrointestinal herbal products; anti-inflammatory and antiviral research	Hairy-root metabolic engineering through targeted overexpression, knockout/downregulation and elicitation	Root-specific biosynthesis and targeted manipulation of biosynthetic regulatory networks	Pathway branching is complex; editing or overexpression may reduce root growth or redirect flux toward unwanted triterpenoids	[35,36]
*Hypericum perforatum* L.	Hypericin; pseudohypericin; hyperforin	Treatment of mild-to-moderate depressive symptoms; antimicrobial research	Elicitation and culture optimisation in cell and organ cultures (mainly shoot cultures); nanoparticle-mediated elicitation as a complementary strategy	Controlled manipulation of light- and stress-responsive naphthodianthrone pathways	Tissue- and light-dependent hypericin and hyperforin accumulation; low or unstable metabolite titres in dedifferentiated cultures; limited reproducibility and scalability	[37,38]
*Panax* *ginseng* C.A. Meyer	Ginsenosides (Rb1, Rg1, Re and related saponins)	Adaptogenic products; immunomodulatory, neuroprotective and metabolic research	Adventitious root and cell-suspension cultures integrated with elicitation and bioreactor cultivation for enhanced ginsenoside production	Controlled ginsenoside production and large-scale biomass propagation under optimised bioreactor conditions	Genotype-, culture system- and elicitation-dependent ginsenoside accumulation; complex pathway regulation limiting predictable optimisation and industrial productivity	[39,40]
*Papaver somniferum* L.	Morphine, codeine, noscapine, papaverine, thebaine and other benzylisoquinoline alkaloids (BIAs)	Analgesic, antitussive and antispasmodic pharmaceuticals	Biotic and abiotic elicitation complemented by pathway engineering through transcription factors and key biosynthetic genes	Increased total alkaloid yield and selective accumulation of benzylisoquinoline alkaloids	Highly branched benzylisoquinoline alkaloid pathway and complex transcriptional regulation limiting predictable metabolic flux; engineering strategies require further validation	[41,42]
*Salvia miltiorrhiza* Bunge	Tanshinones (e.g., tanshinone IIA, cryptotanshinone); salvianolic acids	Cardiovascular and cerebrovascular applications; anti-inflammatory, antioxidant research	Hairy-root cultures combined with elicitation for enhanced tanshinone and phenolic acid production	Root-specific biosynthesis, high genetic stability and efficient production of tanshinones and phenolic acids	Culture condition-, elicitor- and pathway regulation-dependent metabolite accumulation limiting predictable optimisation and industrial scalability	[43,44]
*Scutellaria baicalensis* Georgi	Baicalin, baicalein, wogonin, wogonoside	Anti-inflammatory, antioxidant, antiviral and anticancer applications	Hairy-root cultures for root-specific flavone production, complemented by transcription factor and pathway-guided metabolic engineering	Root-specific flavone biosynthesis, high genetic stability and targeted manipulation of flavonoid biosynthetic pathways	Tissue- and regulator-dependent flavone accumulation; incomplete understanding of pathway regulation and clone-specific variability limiting predictable engineering and large-scale production	[45,46]
*Silybum marianum* (L.) Gaertn.	Silymarin flavonolignans (silybin, isosilybin, silychristin, silydianin)	Hepatoprotective products, liver-disease supportive therapy	Methyl jasmonate elicitation of cell and shoot cultures with combined biotic and nanoparticle-assisted strategies	Enhanced flavonolignan accumulation under controlled culture conditions	In vitro metabolite profiles differing from mature fruits; biomass reduction and intracellular/extracellular partitioning requiring assessment	[47,48]
*Taxus* spp. (especially *T. baccata*/*T. chinensis*)	Paclitaxel, baccatin III and related taxanes	Anticancer therapy	Elicited *Taxus* cell-suspension cultures supported by bioreactor optimisation and genetic engineering of taxane biosynthesis	Industrially established platform for controlled paclitaxel production, enabling year-round cultivation and process optimisation	Slow cell growth, cell-line instability, epigenetic loss of productivity and genotype-dependent elicitor responses limiting long-term industrial performance	[49,50]
*Withania somnifera* (L.) Dunal	Withaferin A; withanolide A; withanone	Traditional adaptogenic use; anti-inflammatory and anticancer research	Elicitation of in vitro shoots and hairy roots (e.g., methyl jasmonate), supported by culture optimisation and pathway-focused approaches	Organ-specific optimisation and controlled elicitor exposure without seasonal limitations	Organ-, chemotype-, dose- and time-dependent withanolide accumulation; higher metabolite titres may reduce biomass	[51]

Abbreviations: BIAs, benzylisoquinoline alkaloids; CBD, cannabidiol; CBG, cannabigerol; TF, transcription factor; THC, Δ9-tetrahydrocannabinol; TIAs, terpenoid indole alkaloids; PAF, platelet-activating factor. Notably, the representative examples compiled in Table 1 reflect the substantial progress achieved in medicinal plant biotechnology. Nevertheless, despite these advances and the remarkable development of the field over recent decades, achieving predictable and efficient enhancement of bioactive metabolite production remains a considerable challenge.

**Table 3:** Examples of carbon nanoparticle-induced modulation of therapeutically relevant secondary metabolite production.

Secondary Metabolite (Phytomedicines)	Plant Species	Elicitor (Carbon Nanotubes, CNTs; Fullerenes; Graphene)	Concentration Associated with a Beneficial Effect *	Concentration Associated with Adverse Effects *	References
rosmarinic acid	*Satureja khuzestanica* in vitro culture (callus)	multi-walled nanotubes	50 μg ml^−1^	500 μg ml^−1^	[113]
caffeic acid	*Satureja khuzestanica* in vitro culture (callus)	multi-walled nanotubes	50 μg ml^−1^	500 μg ml^−1^	[113]
vinblastine	*Catharanthus roseus* in vitro culture (callus)	multi-walled nanotubes	50, 100 mg L^−1^	---	[114]
		graphene	50, 100 mg L^−1^	---	[114]
vincristine	*Catharanthus roseus* in vitro culture (callus)	multi-walled nanotubes	---	---	[114]
		graphene	50, 100 mg L^−1^	---	[114]
flavonoids (total amount)	*Capsicum annuum*	fullerene C60	at 100 and 1000 mg/L under soil moisture of 100 and 75%	---	[115]
	*Solanum lycopersicum*	nanotubes	10, 50, 100, 250, 500, and 1000 mg L^−1^	---	[116]
		graphene	10, 50, 100, 250, 500, and 1000 mg L^−1^	---	[116]
phenols	*Capsicum annuum*	fullerene C60	at 100 and 1000 mg/L under soil moisture of 100 and 75%	---	[115]
	*Solanum lycopersicum*	nanotubes	10, 50, 100, 250, 500, and 1000 mg L^−1^	---	[116]
		graphene	10, 50, 100, 250, 500, and 1000 mg L^−1^	---	[116]
ascorbic acid	*Solanum lycopersicum*	nanotubes	10, 50, 100, 250, 500, and 1000 mg L^−1^	---	[116]
		graphene	10, 50, 100, 250, 500, and 1000 mg L^−1^	---	[116]
charantin	*Momordica charantia*	fullerol [C60(OH)20]	augmented up to 20%	reduction 15%	[117]
insulin	*Momordica charantia*	fullerol [C60(OH)20]	augmented up to 91%		[117]
lycopene	*Momordica charantia*	fullerol [C60(OH)20]	---	reduction 30%	[117]

* Note: The data presented in the *beneficial effect* and *adverse effects* columns reflect the information available in the cited studies. Accordingly, the entries may refer either to the concentrations associated with the reported effects or to the biological responses themselves, depending on how the original results were presented.

## Data Availability

No new data were created or analyzed in this study. Data sharing is not applicable to this article.
